# Effect of nano-hydroxyapatite and ozone on approximal initial caries: a randomized clinical trial

**DOI:** 10.1038/s41598-020-67885-8

**Published:** 2020-07-07

**Authors:** Katarzyna Grocholewicz, Grażyna Matkowska-Cichocka, Piotr Makowiecki, Agnieszka Droździk, Halina Ey-Chmielewska, Anna Dziewulska, Małgorzata Tomasik, Grzegorz Trybek, Joanna Janiszewska-Olszowska

**Affiliations:** 1 Department of Interdisciplinary Dentistry, Pomeranian Medical University, Al. Powstańców Wielkopolskich 72, 70-111 Szczecin, Poland; 2 Department of General and Dental Radiology, Pomeranian Medical University, Al. Powstańców Wielkopolskich 72, 70-111 Szczecin, Poland; 3 Department of Dental Prosthetics, Pomeranian Medical University, Al. Powstańców Wielkopolskich 72, 70-111 Szczecin, Poland; 4 Department of Dental Surgery, Pomeranian Medical University, Al. Powstańców Wielkopolskich 72, 70-111 Szczecin, Poland

**Keywords:** Dental caries, Medical research

## Abstract

The aim of the study was to assess the efficacy of three methods of enamel remineralization on initial approximal caries: (1) a nano-hydroxyapatite gel, (2) gaseous ozone therapy, (3) combination of a nano-hydroxyapatite gel and ozone. Patients (n = 92, age 20–30 years) with initial approximal enamel lesions on premolar and molar teeth (n = 546) were randomly allocated to three groups subjected to a 6-months treatment: Group I: domestic nano-hydroxyapatite remineralizing gel, group II: in-office ozone therapy, group III: both domestic remineralizing gel and ozone therapy. Caries lesions were assessed on bitewing radiographs at baseline, after 1 year and after 2 years. At one-year follow-up, the smallest rate of lesions with remineralisation (36.5%) was found in group I, and the highest (69.3%)—in group III. In group III a significant remineralisation was noticed in after 1 year and then a demineralisation after 2 years. Thus nano-hydroxyapatite gel and ozone therapy exert some capacities to remineralize approximal enamel and dentine subsurface lesions of premolar and molar teeth. Moreover, the combination of both methods produces the best effect compared to nano-hydroxyapatite or ozone therapy applied alone. However, the treatment should be continued for a long time in order to achieve nonrestorative recovery of caries.

## Introduction

Dental caries is considered the most prevalent chronic disease. Recent epidemiological studies revealed a shift in the prevalence of caries with age; in younger subjects carious lesions are more prevalent on occlusal surfaces, whereas with advancing age—on approximal surfaces^1–6^. Approximal non-cavitated caries lesions are difficult to detect directly by visual examination, due to the contact area. Therefore, for early detection and monitoring of approximal caries, clinicians not only perform a visual examination, but also analyse bitewing radiographs for signs of demineralisation^7^. Although some studies indicate that the best method to detect caries on approximal surfaces is the use of a laser fluorescence device or^8–10^, bitewing radiographs are still more popular, since most dental offices are equipped in an X-ray apparatus.

Initial, non-cavitated lesions as well as caries restricted to the enamel (not involving the dentin) can be arrested or even remineralized by applying therapeutic agents^11,12^. Thus this stage of caries is possible to arrest or reverse by modifying the etiologic factors or applying preventive measures. However, the repair proceeds mostly within the superficial layer^13^.

In recent papers, two methods of treating non-cavitated carious lesions could be found. One approach is to remineralize the lesion body with fluoride, casein phosphopeptide amorphous calcium phosphate (CPP-ACP) or nano-hydroxyapatite (nHAP). Hydroxyapatite is the most important part of enamel structure, producing its bright white appearance and eliminating the diffuse reflectivity of light by closing the small pores of enamel surface, it is biocompatible and bioactive^14^. In recent years, nano-materials have been developed, which are capable of remineralizing initial carious lesions of the enamel^15^. Some properties of nano-hydroxyapatite are considered unique compared to hydroxyapatite, e.g. higher solubility, higher surface energy and better biocompatibility^16^.

Another approach is to apply antibacterial agents in order to reduce the level of bacterial species associated to caries and thus to protect the lesion from microorganisms. This is achieved by the use of ozone and chlorhexidine or resin infiltration^17^ as well. Sealants could also be applied for this purpose^18,19^. Ozone has proven to be effective against bacteria (both gram-negative and gram-positive) as well as viruses and fungi^20,21^. It is a strong oxidant, highly bactericidal and thus is used to treat primary root caries or occlusal caries as well as dentinal hypersensitivity^22–26^. Ozone is also capable of remineralizing lesions within the dentin^27^. It opens dentinal tubules within carious lesion (by damaging biomolecules) and, thus enhances remineralization by increasing perfusion of remineralizing agents^28^. The use of ozone in different fields of dentistry has been reported, including: periodontology, endodontics and maxillofacial surgery^29–31^. Some recent papers report the use of ozone for the treatment of initial pit and fissure caries as an alternative to other non-invasive interventions^23,25,29^. Both in vivo and in vitro studies can be found concerning the successful use of ozone to treat caries, disinfect cavities, reduce the level of caries-associated microorganisms in dental plaque or remineralize caries lesions. However, more clinical evidence for ozone application is required before it can be widely used for managing and preventing caries^32^.

The aim of this study was to assess and compare the efficacy of nano-hydroxyapatite gel and gaseous ozone therapy on enamel remineralization of initial caries lesions on approximal surfaces of premolar and molar teeth in adults.

## Material and methods

The study was conducted in years 2012–2016 on 134 participants (96 females and 38 males) aged from 20 to 30 years (median 23.3 years), who were recruited from a pool of patients attending Department of Interdisciplinary Dentistry Pomeranian Medical University in Szczecin, Poland. The characteristic of the study group has been presented in Table 1. The study protocol was approved by the Ethics Committee of the Pomeranian Medical University in Szczecin (KB-0012/102/11) and conducted in accordance with accepted ethical standards for research practice. A written informed consent was obtained from all participants enrolled. The study protocol has been registered in clinicaltrials.gov 30th October 2019 at the reference number NCT04147091.

**Table 1:** Demographic characteristics of study population.

Characteristic	Total (n = 92)	Group I (n = 31)	Group II (n = 30)	Group III (n = 31)
Women	66	26	17	23
Men	26	5	13	8
Age (median)	23.3	23.3	22.6	24.0

The first stage of the study comprised detection of the initial caries enamel lesions on approximal surfaces of premolar and molar teeth (baseline 0), all the patients were subjected to clinical examination followed by X-ray radiographic (bitewing) assessment (Gendex Expert DC, Gendex Dental Systems, USA) and a digital radiography system (Digora 2,5 S, Soredex, Finland). Parameters of X-ray exposition were: voltage—65 kV, anode current—7 mA, X-ray focus—0.4 mm, exposure time—0.08 s. A lead apron was used for radiological protection. In order to obtain repeatability at each examination the radiographs were made using a film holder with an individual positioner made of an occlusal registration material (Futar D, Kettenbach, Germany). The depth of approximal caries lesions was classified according to the scoring system by Mejàre et al.^4^: 0—no visible radiolucency, 1—radiolucency in the outer half of the enamel, 2—radiolucency in the inner half of the enamel, 3—radiolucency in the dentin; broken enamel–dentin border without obvious spread in the dentin, 4—radiolucency with obvious spread in the outer half of the dentin, 5—radiolucency with obvious spread in the inner half of the dentin. Clinical and radiological data were recorded in the study chart. The clinical visual-tactile examination was performed on the dental chair, using a dental probe.

The inclusion criteria for the study were:

- Initial demineralisation lesions not exceeding enamel-dentin junction on approximal surfaces, radiolucency merely within the enamel (score 1 and score 2, not exceeding the enamel–dentine border that were detected on X-ray radiography)
- Existing contact points on approximal surfaces between the premolar and molar teeth

The exclusion criteria were: the distal surface of the last tooth in arch, overlapping teeth, fillings on approximal surfaces of teeth of interest, periodontal disease as well as ongoing orthodontic therapy. All subjects qualified for the study were generally healthy and did not take any medicine. Thus no contraindications for ozone therapy were stated in the study group.

From among 134 examined subjects, 92 were selected (66 women, 26 man) with initial caries on approximal surfaces. At the beginning of the study, all participants received a written information sheet with oral hygiene instructions including tooth brushing method (roll technique minimum twice a day) and flossing (every day after the evening tooth brushing) as well as dietary advice (no more than 5 sugar contacts a day). All patients enrolled reported to have used fluoridated toothpaste (1450 ppm) before the initiation of the study and they got recommendations to continue using such toothpaste during the entire study). Neither the water nor nutritive products were fluoridated.

All participants were then randomly allocated (using random number generator) into three treatment groups. For ethical reasons there was no control group. Sample size was verified using an online power and sample size calculator (powerandsamplesize.com). At the level of clinical significance of 1.5 and power of correlation of 80%, the sample size (number of surfaces assessed) yielded 139.

Group I subjects received a remineralizing gel containing 10% of nano-hydroxyapatite (ApaCare & Repair, Cumdente, Germany) for domestic use (for 6 months daily use). After a thorough evening tooth-cleaning with fluoridated toothpaste and flossing, the gel was applied on the overall teeth using a toothbrush, and the patient abstained from consumption of any food and beverage for a period of 3 h. Each patient was provided with two tubes of the gel.

Group II participants were subjected to ozone therapy carried out 4 times: at first appointment, after 2, 4 and 6 months. Before ozone application, each patient was requested to perform a thorough tooth-brushing and cleansing of approximal surfaces with dental floss. The gas was generated by OzonyTron (Mymed, Germany) apparatus and applied with silicon trays for both tooth arches simultaneously, using the full mouth disinfection program for 5 min. This procedure was performed in all patients by the same experienced operator (the second author).

Group III subjects received a combination of the remineralizing gel for domestic use and ozone therapy in the same treatment courses.

The period of treatment was 6 months in every group and the follow-up examinations were carried out after 1 year (follow-up 1), as in the studies by Ekstrand et al.^33^ as well as by Arslan and Kaplan^34^ and after 2 years (follow-up 2) from the beginning of the study. The clinical examination on control visits was only adjunct to radiographic assessment; progression, regression as well as an absence of demineralization lesions were analysed in bitewing radiographs by the blinded second author. The primary outcome was caries lesion progression or regression.

To determine the optical density (pixel intensity = gray value) in the central area of caries lesion the density measurement tool in Digora 2,5S Software was used. A point located within the dentin in half distance between cemento-enamel junction and pulp cavity wall was determined and used as a reference for measuring optical density of the cavity. Then the difference between the lowest value of optical density within the cavity and the density of the reference point located within the dentin in half distance between the cemento-enamel junction and pulp cavity wall was calculated. Comparing the value of the differences in optical density between the lesion area and the reference point in subsequent examinations allowed to determine whether there is regress or progress or no change in optical density of the analysed area. A reduced difference indicated increasing density in the lesion area, which meant caries regression.

The Statistica 12 Pl version software was used for data analysis. During the evaluation of the study data, along with the descriptive statistical methods (frequency), the quantitative variables that did not have a normal distribution were analysed using the Mann–Whitney U test and the Wilcoxon signed-rank test was used to compare two dependent samples. Data related to optical density, differences in optical density between two areas were analysed using the Mann–Whitney U test and Wilcoxon signed rank tests, for intergroup and intragroup comparison, respectively. A significance level was set at p < 0.05 for all statistical tests.

## Results

**Figure 1:**
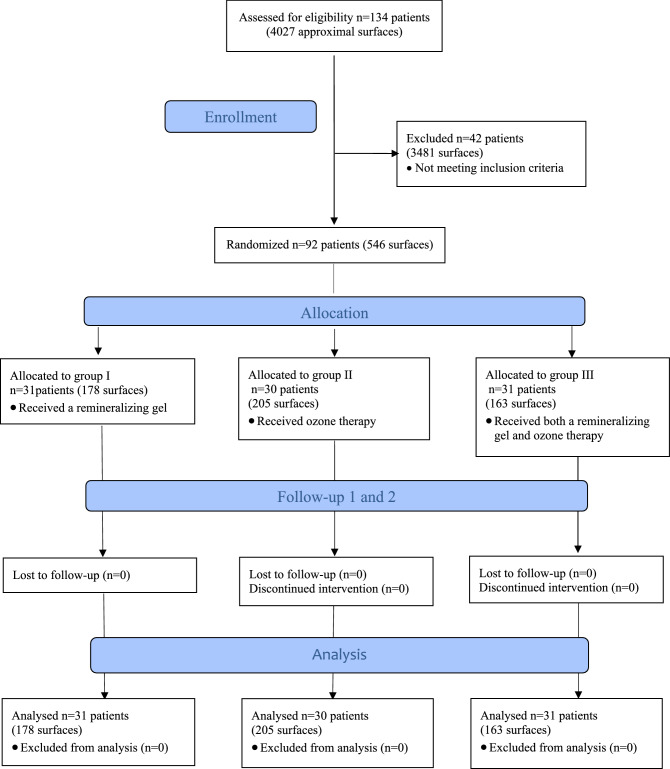
CONSORT flow chart of the study.

At the beginning of the study, 4,027 approximal surfaces of premolar and molar teeth in 134 patients were examined (Fig. 1). Out of them, 3,481 did not meet the inclusion criteria and thus were excluded from the study. The study design flow is depicted in Fig. 1. The caries lesions observed in clinical evaluation on 48 approximal surfaces received radiological examination scores higher than 2 and required invasive treatment, remaining 3,433 surfaces were scored 0. Finally, a total of 546 initial enamel lesions (score 1 and 2) in 92 patients were subjected to observation and none of them was detectable during visual clinical examination. From the 546 approximal surfaces, 307 were in molars and 239 in premolars.

Table 2 presents differences between average optical densities in the lesion area and the reference point in subsequent examinations in all observed groups. In group I applying nano-hydroxyapatite gel the mean values of differences between reference point and area analyzed increased in follow-ups relative to baseline, indicating a progress of demineralisation. In group II (subjected to ozone therapy) the difference of optical densities in two compared areas after one year slightly decreased (indicating remineralization) and after two years increased. In group III (provided with nano-hydroxyapatite and ozone therapy) in follow-up 1 a significant decrease (remineralization) was noticed and then in follow-up 2 an increase (demineralisation) of differences between optical densities. Significance levels of optical density differences in compared groups in subsequent examinations are presented in Table 3.

**Table 2:**
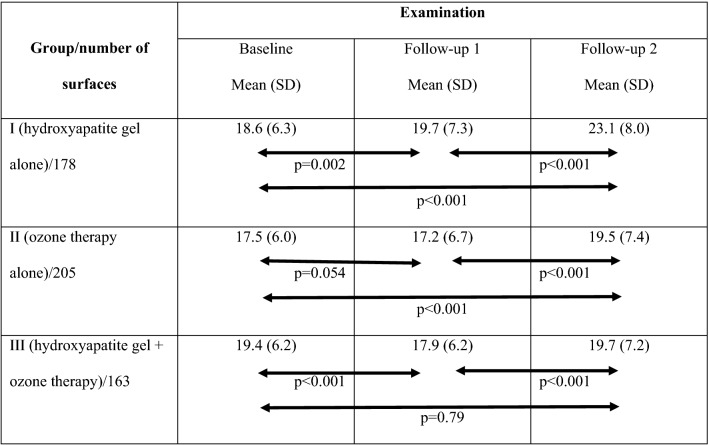
Differences between average optical densities in the lesion area and the reference point in subsequent examinations.

**Table 3:** Significance levels of optical density differences in compared groups in subsequent examinations.

Compared groups	Examination		
	Baseline	Follow-up 1	Follow-up 2
I vs. II (178/205)	NS (0.12)	0.002	< 0.001
I vs. III (178/163)	NS (0.23)	0.073	< 0.001
II vs. III (205/163)	0.005	NS (0.24)	NS (0.61)

After 1 year of the observation (Table 4) in group I a regression of initial caries lesions was found in 36.5% lesions analyzed. In group II regression was stated in 60% lesions. In group III a regression was found in 69.3% of analysed lesions.

**Table 4:** Changes of optical density differences in particular groups—results after 1 year (baseline vs. follow-up 1).

Optical density difference	Group						p*		
	I		II		III				
	*n*	%	*n*	%	*n*	%	I vs. II	I vs. III	II vs. III
Decrease	65	36.5	123	60	113	69.3	< 0.001	< 0.001	0.08
No change	9	5.1	19	9.3	9	5.5	NS (0.17)	NS (0.96)	NS (0.25)
Increase	104	58.4	63	30.7	41	25.2	< 0.001	< 0.001	NS (0.29)

*p < 0.05 significant.

After two years of the study (Table 5) the findings in particular groups were similar to those at the one-year follow-up. The smallest rate of caries reversal was observed in group I (18.0%). In group II it was 38.0%, and in group III 45.4%.

**Table 5:** Changes of optical density differences in particular groups—results after 2 year (baseline vs. follow-up 2).

Optical density difference	Group						p*		
	I		II		III				
	*n*	%	*n*	%	*n*	%	I vs. II	I vs. III	II vs. III
Decrease	32	18.0	78	38.0	74	45.4	< 0.001	< 0.001	NS (0.19)
No change	9	5.1	15	7.3	16	9.8	0.48	NS (0.14)	NS (0.50)
Increase	137	77.0	112	54.6	73	44.8	< 0.001	< 0.001	NS (0.08)

*p < 0.05 significant.

## Discussion

The present study showed that nano-hydroxyapatite gel application and ozone therapy have some potential to remineralize enamel lesions, but a combination of both methods produces the best effect. Observation of changes in particular groups revealed that the differences between average optical densities in two compared areas decreased the most in group provided both preventive agents, pointing to the best therapeutic effect. Six months after treatment cessation (follow-up 1) optical density was significantly higher than one year later (follow-up 2).

Concerning to restorative and preventive aspects, nano-hydroxyapatite exerts a significant remineralizing effect on initial enamel lesions, which is certainly higher than of the traditionally used fluorides^14^.

An In vitro study comparing nHAP dentifrice and a fluoride dentifrice (5% nHAP, 10% nHAP, 1,100 ppm fluoride) showed that remineralization achieved was similar, and inhibited caries development, thus suggesting that an nHAP dentifrice can be an effective alternative to fluoride toothpaste^35^. Tschoppe et al.^36^ analyzed the remineralizing effect of toothpastes containing n-HAP compared to amine fluoride toothpastes with bovine dentine. Their study revealed that toothpastes containing nano-hydroxyapatite of different types showed a similar remineralizing potential to enamel and dentin, however toothpastes enriched in fluoride had lower properties than the aforementioned. In another study, Huang et al.^37^, analysed the effect of four nano-HAP concentrations on initial enamel caries lesions in cyclically varying pH and confirmed that nano-hydroxyapatite is able to remineralize enamel. Moreover at each time point in the pH-cycling, different concentrations were exerted distinct effects on remineralization and an optimal concentration of nano-hydroxyapatite for enamel remineralization proved to be a 10% suspension. The authors concluded that nano-hydroxyapatite used daily in a proper concentration could be beneficial in promoting remineralization. Hill et al.^38^ in their in vitro study demonstrated a higher dense particle coverage and occlusion of the dentin tubule by nHAP oral rinse compared to the zinc-substituted hydroxyapatite. El Assal et al.^39^ proved that nano-hydroxyapatite, especially when combined with application of a laser, had a significant remineralizing effect on initial enamel lesions, and that the pure type of nHAP shoved a higher effect than the nano-fluoroapatite type.

All of the above studies indicating the efficacy of nHAP in remineralizating initial caries were carried out in vitro, whereas the present study revealed remarkable remineralizing effects of this agent in vivo.

Atabek and Oztas^40^ proved that ozone both alone and combined with a remineralizing agent is effective in caries reversal of initial fissure lesions. Samuel et al.^41^ carried out an in situ study on artificially created subsurface demineralization and concluded that ozone (ozonized water) significantly improved the remineralizing capability of nano-hydroxyapatite.

Few studies have been published referring to the effect of ozone on enamel remineralization. No clinical studies could be found in the literature, to investigate the effect of ozone application combined with nano-hydroxyapatite on approximal initial caries as was the interest of the present investigation. In our study we observed that ozone had a potential of initial caries reversal. However, major effect was noticed when it was combined with a remineralizing adjunct: nano-hydroxyapatite. The results of our study are in agreement with the observations by Samuel et al.^41^, who showed that ozone significantly improved the remineralizing capability of nano-hydroxyapatite on artificially induced enamel caries lesions compared to nano-hydroxyapatite alone. Results contrary to the present study were reported by Tahmassebi et al.^42^, who analysed in vitro the effect of ozone on artificial caries-like lesions and concluded that ozone alone is not effective in promoting remineralization or preventing enamel demineralization unless combined with an agent containing high levels of fluoride. The diverse observations may result from a different way of ozone application and a different ozone generator. Moreover, the effect of ozone on the reduction of bacteria in the lesion could not be studied in vitro. Yazıcıoğlu and Ulukap^17^ in their study applied ozone in vivo from different generators and used different treatment courses than in the present study. They concluded that the ozone gas application inhibited the demineralization activity due to the microorganism removal merely in the outer half of enamel. This finding is similar to our conclusion referring to the regression of lesions in the study group that underwent ozone treatment alone. Atabek and Oztas^40^ studied the effect of ozone alone and combined with a remineralizing solution on early fissure caries in permanent molars and concluded that applying ozone, either alone or with a remineralizing solution, can lead to caries reversal, since ozone therapy alone in correct oral conditions promotes remineralization.

It should be noticed that all the patients involved in the present study used fluoridated toothpaste for daily oral hygiene before, during and after the treatment. Thus fluoride could be an additional factor enhancing remineralization. This factor was uniform in all the study groups.

Possible limitations of the present study are the facts that the patients were not blinded to the treatment and application of nanohydroxyapatite gel was based on trust (its use could not be objectively verified).

In conclusion, the results of the current study indicate that nano-hydroxyapatite gel and ozone therapy exert some properties to remineralize initial approximal enamel and dentine subsurface lesions of premolar and molar teeth. The combination of both methods produces the best effect compared to nano-hydroxyapatite or ozone therapy alone. The treatment procedures should be continued for a long time in order to achieve the effect of nonrestorative treatment of caries.
